# Invited discussant comments during the UCL–Penn Global COVID Study webinar ‘Reflections, Resilience, and Recovery: A qualitative study of Covid-19’s impact on an international adult population’s mental health and priorities for support’: part 3 of 3

**DOI:** 10.14324/111.444/ucloe.100007

**Published:** 2022-12-01

**Authors:** David Murphy

**Affiliations:** 1 Professor of Clinical Psychology, School of Psychology, Faculty of Health, University of Plymouth, Plymouth, Devon, PL4 8AA, UK; 2 2019–2020 President, British Psychological Society

**Keywords:** psychological, people, support, Covid-19, older, old age, ageing, benefits, pensions, poverty, pandemic, loneliness, mental health, physical health, health, wellbeing

## Abstract

This discussant commentary considers the findings presented from the UCL-Penn Global COVID Study webinar ‘Let’s Talk! What do you need to recover from Covid-19?’ and published in Wong et al’s article in this journal, Reflections, Resilience, and Recovery, drawing into focus the support required to recover from the changes in people’s mental health, physical health and relationships brought on by the Covid-19 pandemic. Acknowledging the importance of not making broad generalisations about the effect of the lockdown allows us to see individuals in their own context and their own particular challenges. As we emerge from the Covid-19 pandemic, we need to use the lessons from this study as the foundations for building resilience against future pandemics.

**About the study**

The UCL-Penn Global COVID Study, launched in April 2020, is a 12-month longitudinal study of the impact of Covid-19 on social trust, mental health and physical health. The Covid-19 global pandemic can be seen as a ‘natural stressor’ or major change in the environment that allowed researchers to study how changes in the environment can have an impact on individuals’ relationships with others and their health. In collaboration with six institutions from Italy, Singapore, the USA, China and the UK [1], the study looks at the short- and longer-term effects of Covid-19 on individuals’ mental health and social relationships with others. Survey data was collected at three timepoints: 17 April to 14 July 2020 (wave 1), 17 October 2020 to 31 January 2021 (wave 2), and 17 April to 31 July 2021 (wave 3).

**About the webinar**

Held online between 2 June and 28 July 2021, the study group presented study data at five online webinars as part of the UCL Global Engagement Fund sponsorship, to discuss the lessons learned. Policymakers and other subject experts were invited to speak on the policy relevance and implications of the study findings. A conscious decision was made by the study team to situate the study findings of individual’s health and relationships in the context in which they occur – local communities and countries. The recorded comments from these discussions, focusing on the policy relevance and implications of each academic article, were recorded as discussant articles and published in this journal to be read alongside the research article being discussed.

These discussant articles are reviewed by members of the Editorial Board before being published. It is hoped that these discussant articles, read alongside the research articles, will provide more holistic understanding of the issues at hand, how findings may inform policies in the coming months and/or assist in future cisis management strategies and aid decision-making, in an open and transparent manner.

The study was pre-registered (https://osf.io/4nj3g/ on 17 May 2021) and ethical approval was obtained from the IOE (Institute of Education), UCL’s Faculty of Education and Society (University College London, UK) Ethics and Review Committee on 8 April 2020 (REC 1331) [2].

**Linked research article**

The linked research article to this discussion article cited here has been published in *UCL Open: Environment* following open peer review and made freely available to read as an open access article. Additionally, all previous versions and peer review reports are freely available to read as open access preprint articles from the journal’s website by following the below DOI link and navigating to the version history of the published research article. Readers can find more information about how peer review works in the journal at ucl.scienceopen.com.

Wong KK, Loke K, Melville KMK. Reflections, Resilience, and Recovery: A qualitative study of Covid-19’s impact on an international adult population’s mental health and priorities for support. *UCL Open: Environment*. 2022;(4):12. DOI: https://doi.org/10.14324/111.444/ucloe.000041.

**Recorded webinar**

This discussion article comments on the findings of the research article presented during the following webinar that has been recorded and made freely available to readers to watch on-demand.

Summer Webinar 5 - Let’s Talk! What do you need to recover from COVID-19? #GlobalCOVIDStudy. Available from: https://www.youtube.com/watch?v=i8z9KzIIcj0.

## Discussant comments

The Covid-19 pandemic has been like a huge stone dropped into a still pond. A sudden shock and then a series of ripples that spread far out and stay for a very long time. Firstly, of course, at the time of writing, there are 129,000, and still rising, people who have sadly died in England [3]. These deaths were very unevenly distributed; from April to July 2020, a staggering 75% of all Covid-19-related deaths in England were among people in care homes [4] – predominantly the elderly, but also people with intellectual disabilities. The mortality rate was also dramatically different for people from different ethnic backgrounds. In England, those from Asian/Asian British backgrounds were nearly three times more likely to die than those from White/White British backgrounds, and those from Black/Black British backgrounds twice as likely [3].

The less well-documented side of those 129,000 deaths is the hundreds of thousands of bereaved husbands, wives, children and sometimes parents and friends. Many were unable to be with their loved one at the time of their death or be supported in-person themselves by family and friends in the aftermath.

Whilst the vast majority of those infected with coronavirus did survive, a significant proportion continue to experience symptoms and disability; there are up to a million people in the UK alone with long Covid [5]. Many others, whose health was not directly affected by Covid-19, have been indirectly affected by lack of access to physical activity and limitations on, and reluctance to use, usual healthcare services.

Health and social care staff have been working under intense pressure over the past 18 months, many of who were transferred to different roles in unfamiliar settings. The backlog in routine National Health Service (NHS) care means that the pressure on NHS staff will continue for years to come.

There have also been wider impacts on particular parts of society, and especially on younger people whose education has been impacted and whose social development and transitions from school to university have also been disrupted. Many adults have been struggling to adapt to working at home and also adapting to home schooling as well, with others experiencing isolation and loneliness. During lockdown many people, predominantly women, also suffered from the rise in domestic violence.

Thus, although severe acute respiratory syndrome coronavirus 2 (SARS-CoV-2) is a tiny virus that we have to look under a microscope to see, its effects have been really very far-reaching, and will sadly persist for many years.

However, I do also think there have been some positive things to come out of the pandemic. The extent and speed of interprofessional and international collaboration has been incredible. The UCL–Penn study is certainly an example of this, launching the first wave in mid-April last year, and recruiting a large, international sample is really impressive.

There are a few caveats to the study that need to be borne in mind when interpreting the results. Clearly any study that recruits online advertising is likely to reach an unrepresentative sample, and within that sample some people are more likely than others to opt in, thus creating a selection bias. It is therefore unsurprising that this sample is more highly educated with a higher income than average population in the country studied.

Furthermore, in this longitudinal study over three points of time, there were some people who participated at just one point. Therefore, we need to be mindful that differences between timepoints may reflect differences in the sample at the different points rather than overall changes over time.

One further area of caution relates to interpreting the answers about self-reported needs. Participants’ responses describing their own emotional state obviously have a high validity as they are best placed to comment on their own state. However, we need to be more cautious when making statements about what would help them, as that also requires knowledge of evidence based on different interventions.

Something we all heard a lot at the beginning of the pandemic was that ‘we’re all in this together, we’re going through the same storm!’. I think that what is clear from study findings and the comments of the other discussants, is that whilst we may have been through the same storm, we have been in different boats. We have all lived through a pandemic, but some people’s experience of the pandemic has been very different to others. The experiences during lockdown of a single parent, living in a flat in a high-rise tower block and having to home school young children, was very different to a middle-aged couple living in a spacious house with a garden. Therefore, a theme that comes out from this, and other, studies of the pandemic is the importance of not making broad generalisations about the effect of lockdown, but to see individuals in their own context and their own particular challenges and resources.

What also comes out very clearly from the pandemic and this study is the importance of connections. We are connected to each other, and we are connected in different ways to a wider circle than perhaps we realised pre-pandemic. There is also the potential for greater connection with people in a local community. Over the past 18 months, I have often thought about John Donne’s 400-year-old poem, ‘Meditation 17’, which includes the famous line: ‘No man is an island, entire of itself; every man is a piece of the continent, a part of the main’. We often take pride in our imagined independence, but the Covid-19 pandemic has shown us how dependent we really are on the supermarket workers, on the transport workers, on the refuse collectors around us. Moreover, whilst ‘no man is an island’, no *island* is truly an island. Being an island nation, and recently leaving the European Union (EU), we are more at risk of focusing exclusively within our own borders, but the pandemic has demonstrated in stark terms that we are all connected. As the World Health Organization (WHO) Director-General, Dr Tedros Adhanom Ghebreyesus, implored recently in relation to vaccination, ‘nobody is safe until everybody is safe’.

I think the other theme that really comes across is the importance of social support and the value of family and friends. Some of the participants talked about professional mental health services, and clearly these are important and have been facing significant demand. However, also important is facilitating support from friends, family and colleagues. It is something we have focused a lot on when considering NHS staff wellbeing – trying to put things in place to facilitate people supporting each other because that is our first line of support. Many of us watched the Olympic Games in Japan this summer, and although we often think of Olympic athletes as somehow immune to the things that affect most people, it has been striking to see how many have spoken about the impact on their performance of not having family and friends around them. So, if it affects even Olympic athletes, it will certainly impact the rest of us.

Therefore, I think the importance of facilitating social support and also managing anti-social behaviour and bullying, which another participant during the presentation also highlighted, has certainly come out from the pandemic and the study as a significant factor.

Drawing out these themes leads naturally to thinking about applying them in the recovery from the pandemic, to ‘build back better’. Although this term has been used a great deal during the pandemic, it actually predates it by some years. The term was officially used for the first time in 2015 with the development of the United Nations disaster reduction plan [6]. The concept was put forward by the Japanese delegation who were hosting that conference in Sendei. Just four years earlier in Japan, the Fukushima nuclear power disaster had been triggered by a 15 meter tsunami following a major earthquake and their aim through the recovery was to build greater resiliency against possible future disasters. To truly build back better, we need to recognise the value of community and work to foster this, including recognising our dependencies on others. We should look outwards and be open to learning from others and working to foster collaboration and community.

As we emerge from the Covid-19 pandemic – and we are still certainly not out of it yet – we need to use the lessons from this, and other studies, as the foundations for building resilience against future pandemics, and also for learning wider lessons about more everyday sources of challenges and stresses.
